# Rural-urban Differences in Sociodemographic, Social Network and Lifestyle Factors Related to Mortality of Middle-aged Japanese Men from the Komo-Ise Cohort Study

**DOI:** 10.2188/jea.12.93

**Published:** 2007-11-30

**Authors:** Motoki Iwasaki, Tetsuya Otani, Akiko Ohta, Sasazawa Yosiaki, Masaya Kuroiwa, Shosuke Suzuki

**Affiliations:** Department of Public Health, Gunma University School of Medicine.

**Keywords:** rural-urban differences, sociodemographic factors, lifestyle, social networks, mortality

## Abstract

To examine rural-urban differences in the relationships of sociodemographic, social network, and lifestyle factors to mortality in middle-aged men, we used the data from a community based prospective cohort study, the Komo-Ise study. The subjects were all men aged 40-69 years living in Komochi Village, the rural group (n=2,295), or the downtown district of Isesaki City, the urban group (n=3,334), as of 1993. They completed a self-administered questionnaire in 1993 and were followed for all-cause deaths until 2000. The Cox proportional hazards model was used to compute relative risks (RRs) with 95% confidence intervals (CIs). Low educated men and men without a spouse in the rural group had an increased risk of mortality (RR=4.4; 95%CI: 1.1-18.2, RR=2.4; 95%CI: 1.2-4.5). Men who did not enjoy good fellowship with their neighbors in the rural group had a decreased risk of mortality (RR=0.58; 95%CI: 0.35-0.97). Mortality risks were significantly higher in urban men not participating in hobbies, club activities or community groups (RR=1.6; 95%CI: 1.1-2.4). These variables remained significant risk factors, even after controlling for all sociodemographic, social network, lifestyle, and health status variables. Educational level, marital status and relation to neighborhoods showed significant rural-urban differences.

## INTRODUCTION

The dramatic shift in the major causes of disability and death from infectious to chronic diseases has made theories of disease etiology shift from a single factor to multiple factors including behavioral and environmental as well as biologic and genetic factors. Among these factors, many epidemiologists have focused on health behavior or lifestyle factors, such as smoking habit, overweight, alcohol consumption, physical inactivity and so on, for the past several decades^1^^-^^4^^)^. A number of prospective studies based on randomly selected cohort groups have indicated these to be the major determinants of premature and preventable disease and/or death^1^^-^^4^^)^. In addition to health behavior and lifestyle factors, poor social networks and social support have been recognized as independent risk factors for mortality^5^^-^^7^^)^, since Cassel^8^^)^ hypothesized that psychosocial effects in the environment increased a person’s resistance to risk factors.

Among the earlier large investigations of the relationship between health and human ties, was the study performed in Alameda County, California. Berkman et al.^5^^)^ examined four social network sources: 1) marriage; 2) contacts with close friends and relatives; 3) church membership, and 4) informal and formal group associations. Their findings revealed social and community ties to be associated with mortality risk and each of the four sources to be a predicted risk factor for mortality independently from the other three. However, the Tecumseh Community Health Study^9^^)^ and the Evans County Cardiovascular Epidemiologic Study^10^^)^ results were not consistent with those of the Alameda County Study. The Tecumseh Community Health Study^9^^)^ replicated and extended Berkman’s work in the Alameda County Study. House et al.^9^^)^ reported only men with higher levels of social relationships and activities to be significantly less likely to die during the follow-up period. Notable differences were somewhat weaker associations of mortality with friend and relative contact and religious involvement, and the much stronger association of mortality with other organizational involvements in the Tecumseh community as compared to those in the Alameda County Study. The Evans County Cardiovascular Epidemiologic Study^10^^)^ also replicated Berkman’s study of social networks and mortality in Alameda County. Schoenbach et al.^10^^)^ found evidence of a relationship between social networks and mortality only among white males and that the social network effects among white females, black males, and black females were weaker and clearly non-significant. These findings suggested social networks to have different meanings and effects according to locality^7^^)^. Seeman et al.^11^^)^ hypothesized that previous inconsistencies in the findings from different studies might indeed reflect sociocultural differences among the various samples based on a comparative analysis of three communities for elderly people.

In a number of subsequent studies, not only social networks but also social supports constituting social relationships have increasingly been recognized as risk factors for mortality and morbidity^12^^,^^13^^)^. In addition to social support, the effect of stress on mortality has been analyzed simultaneously^14^^)^. Social relationship studies have been designed and conducted. Large amounts of interesting knowledge have thus been accumulated for many years. Nonetheless, the impact of intercommunity variations on the association between social networks and mortality in middle-aged people remains unknown.

We have accumulated little knowledge on the association between social networks and mortality among middle-aged Japanese men, especially rural-urban differences in mortality risk. We conducted a community based prospective study, the Komo-Ise Study, that began with a baseline sample in 1993 for the purpose of clarifying the association between sociodemographic, social network, and lifestyle factors and mortality in middle-aged residents of Gunma prefecture. Some notable results have already been reported from baseline data^15^^-^^17^^)^. Herein, the purpose of this study is to clarify rural-urban differences in the relationships of sociodemographic, social network and lifestyle factors to mortality in middle-aged men. We report the results, focusing especially on social network factors.

## MATERIALS AND METHODS

### Study population

The Komo-Ise Study includes two research areas. One is Komochi Village which is located in the center of the Japan Archipelago, in the northwest comer of the Kanto Plain. The other is Isesaki City which is located in the southeast of Komochi Village. The village had a population of 12,141 with 3,284 households and the city had a population of 120,236 with 40,335 households, according to the 1995 census^18^^)^. All male and female residents aged 40 to 69 years, 4,875 in Komochi Village and 7,755 in the downtown district of Isesaki City, were selected from registration records. In this study, “downtown district” denoted former Isesaki Town historically that has been the center of Isesaki City for more than 50 years. Self-administered questionnaires were distributed through the respective municipal government offices to all of Komochi Village in January 1993 and to those of the downtown district of Isesaki City in October 1993, and completed questionnaires were collected in sealed envelopes. A total of 11,565 subjects in both areas responded to the questionnaire (response rate: 91.6%) in the baseline survey. Ohta et al.^17^^)^ have already reported that non-response bias and selection bias were inconsiderable in this baseline study. The present study analyzed data from all men aged 40-69 years living in Komochi Village (n=2,295, a rural group) or the downtown district of Isesaki City (n=3,334, an urban group).

### Study variables

The questionnaire consisted of items on sociodemographic characteristics, social network, lifestyle, health status, and a symptom checklist known as the Todai Health Index (THI)^19^^,^^20^^)^.

Sociodemographic items included sex, age, occupation, and educational background. Respondents were asked about the longest-held occupation, not employment status in this study. They were grouped into the following four categories: salaried workers; self-employed workers; agriculture and forestry; and no occupation. Educational background was coded into dichotomous categories: compulsory education, high school, vocational school or special school, versus junior college and college or higher.

Social network items encompassed seven factors in the present study, that is, marital status (Marriage), household size (Household), number of meeting close relatives (Relatives), having reliable friends (Friends), participation in activities (Participation), going to any religious services (Religion), and enjoying good fellowship with neighbors (Neighborhood). Respondents were asked the following questions to assess each social network item: 1) “What is your current marital status: married, single, divorced, or widowed?”; 2) “How many people do you live with?”; 3) “How often do you meet close relatives not living with you: almost daily, more than ten times a month, 5 to 9 times a month, one to 4 times a month, a few times a year or rarely or never?”; 4) “Do you have any close friends in need: yes or no?”; 5) “How often do you take part in hobbies, club activities or community groups: very often, often, sometimes, or never?”; 6) “Do you go to any religious services: yes or no?”; 7) “Do you enjoy good fellowship with your neighbors: yes or no?” These seven items were coded into dichotomous categories, except for household size (Household). Marital status (Marriage) was categorized into married and others: single, divorced, or widowed. Number of meeting close relatives (Relatives) combined these responses: almost daily, more than ten times a month, 5 to 9 times a month and one to 4 times a month into one category. Participation in activities (Participation) combined these responses: very often, often, and sometimes in one category.

Lifestyle and health status items included smoking habit, alcohol consumption, physical activity, BMI (body mass index: weight (kg)/height (m)^2^), chronic diseases, and the depression scale of the Todai Health Index (THI). Smoking habit was coded as never smoked, former smokers, and current smokers. Alcohol consumption was assessed by asking, “Do you drink a lot of alcoholic beverages: yes, drink a little, or never?” Responses were coded into dichotomous categories: yes versus drink a little and never. Physical activity was assessed by asking “Do you exercise regularly: often, sometimes, or never?” Responses were coded into dichotomous categories: often and sometimes, versus never. BMI, calculated from self-reported data, was coded into three categories: less than 18.5, 18.5 to less than 25.0, and 25.0 or more. Respondents were also asked whether they had any chronic diseases. The THI consists of 130 questions about subjective physical and psychological symptoms^19^^)^. Each question has three possible responses (affirmative, negative, neutral). The THI has 12-scaled scores and three discriminant functions that are calculated on the basis of the scaled score. The subjective physical symptoms and psychological complaints (130 items) addressed by the questionnaire are classified into those 12 scales, derived by means of factor analysis. A given scaled score is correlated with the frequency of physical symptoms and psychological complaints. The depression scale of the THI was established for use in epidemiologic surveys and monitoring the severity of the depressive state, by testing its content and cross validity in relation to the Center for Epidemiologic Studies Depression Scale and the Zung Self-rating Depression Scale^20^^,^^21^^)^.

Previous investigations have found these sociodemographic factors, lifestyle factors, health status and depression, to be related to social networks and mortality^22^^)^. Thus, these are included in the analysis primarily as a means of controlling for potential confounders.

### Follow up procedures

Information on deaths and migrations in the cohort study was obtained from the municipal resident registration file, *Jumin Kihon Daicho*, from 1993 to 2000 in each area. The cause of death for deceased cases was obtained from death certificates, *Shibo-Kohyo*, in the local public health center with permission from the Management and Coordination Agency, the Government of Japan. All-cause mortality was used as the main outcome variable, though underlying causes of death obtained from death certificates were categorized according to the International Statistical Classification of Diseases, 10^th^ Revision (ICD-10). We added a mail inquiry as a means of reaching subjects who had migrated out of the study area. Subjects who did not respond to the mail inquiry or who had not been reached were regarded as censored cases^17^^)^. The observation period for one subject in each area had not been confirmed in spite of careful follow-up survey (n=2,294, the rural group, and n=3,333, the urban group).

### Statistical methods

Prior health status was thought to have the largest effect during the early follow-up period, as those already ill at the time of the baseline study may be more isolated and have a higher probability of death. Thus, in order to reduce the possible effect on mortality due to unreported or unrecognized health conditions at baseline, cases dying within the first year were excluded from this analysis.

The Cox proportional hazards model was used to estimate relative risks (RRs) with 95% confidence intervals (CIs) for assessing the associations of sociodemographic, social network, and lifestyle factors with all-cause mortality. Age-adjusted relative risks were computed for each area, and multivariate relative risks adjusted for age, occupation, educational background, marital status (Marriage), household size (Household), number of meeting close relatives (Relatives), having reliable friends (Friends), participation in activities (Participation), going to any religious services (Religion), and enjoying good fellowship with neighbors (Neighborhood), smoking habit, alcohol consumption, physical activity, BMI, chronic diseases, and depressive status were calculated for each area.

Analyses were conducted using SPSS version 10.0J for Windows.

## RESULTS

Table 1 shows the baseline data from the rural and urban groups which were already excluded the first year deaths. The mean ages of the rural and urban subjects were 52.6 and 54.8 years, respectively. The proportion of younger subjects was greater in the rural group and approximately 20% worked in agriculture and forestry. On the other hand, the age distributions were nearly identical and about 40% were self-employed in the urban group. In addition, a large proportion of urban subjects was high educated. The two groups were quite similar in marital status (Marriage) and participation in activities (Participation). Household size (Household) tended to be smaller in the urban than in the rural group. The proportion of subjects who often or sometimes met close relatives (Relatives) was 68.6% in the rural and 71.7% in the urban group. The proportion of subjects with reliable friends (Friends) was 63.5% in the rural and 56.2% in the urban group. The proportion of subjects going to any religious services (Religion) was only 3.7% in the rural and 6.1% in the urban group. The proportion of subjects enjoying good fellowship with neighbors (Neighborhood) was 39.5% in the rural and 32.9% in the urban group. The proportions of current and former smokers were 59.4% and 15.8%, respectively, in the rural group, and 56.2% and 17.5%, respectively, in the urban group. The proportion of heavy drinkers was 27.6% in the rural and 25.9% in the urban group. The proportion of subjects who reported that they often or sometimes exercised was 40.0% in the rural and 49.6% in the urban group. The proportions of rural and urban subjects in the BMI category of 18.5 to less than 25 were 75.1% and 73.1%, and the proportions with a BMI of less than 18.5 were 3.1% and 4.8%, respectively.

**Table 1.** Baseline sociodemographic, social network, and lifestyle factors, and health status in Komochi Village (rural) and the downtown district of Isesaki City (urban).

Variable or characteristics	Rural				Urban			
	
	No. of subjects	%	No. of deaths	%	No. of subjects	%	No. of deaths	%
Age								
40-49 years	968	42.4	18	13.7	1018	30.7	21	10.1
50-59 years	700	30.7	35	26.7	1124	33.9	57	27.5
60-69 years	614	26.9	78	59.5	1173	35.4	129	62.3
Occupation								
Salaried worker	1129	52.1	44	35.8	1827	56.5	115	58.1
Self-employed	522	24.1	37	30.1	1232	38.1	65	32.8
Agriculture and forestry	467	21.5	36	29.3	25	0.8	2	1.0
No occupation	50	2.3	6	4.9	147	4.5	16	8.1
Education								
Junior college and college or higher	261	11.9	3	2.5	795	25.1	36	18.3
Except above	1929	88.1	119	97.5	2377	74.9	161	81.7
Marriage								
Married	1819	87.4	94	82.5	2757	86.2	170	84.2
Except above	263	12.6	20	17.5	441	13.8	32	15.8
Household								
Alone	48	2.2	3	2.4	158	4.9	13	6.4
2	363	16.3	38	29.9	831	25.8	78	38.4
3	459	20.6	22	17.3	781	24.3	56	27.6
4	481	21.6	21	16.5	676	21.0	32	15.8
5	357	16.1	11	8.7	404	12.6	10	4.9
6	300	13.5	17	13.4	260	8.1	8	3.9
More than 7	216	9.7	15	11.8	109	3.4	6	3.0
Relatives								
Often, sometimes	1099	68.6	78	72.9	1770	71.7	127	74.3
A few, never	502	31.4	29	27.1	698	28.3	44	25.7
Friends								
Yes	1393	63.5	77	64.2	1797	56.2	111	56.1
No	800	36.5	43	35.8	1398	43.8	87	43.9
Participation								
Yes	1582	72.0	73	61.9	2338	72.9	124	62.9
No	616	28.0	45	38.1	869	27.1	73	37.1
Religion								
Yes	82	3.7	7	5.6	199	6.1	14	6.9
No	2112	96.3	117	94.4	3067	93.9	189	93.1
Neighborhood								
Yes	870	39.5	69	55.6	1059	32.9	70	35.5
No	1334	60.5	55	44.4	2164	67.1	127	64.5
Smoking habit								
Never	549	24.8	19	15.1	851	26.3	47	23.6
Former	349	15.8	34	27.0	567	17.5	26	13.1
Current	1315	59.4	73	57.9	1820	56.2	126	63.3
Alcohol consumption								
Never, light	1602	72.4	85	70.2	2387	74.1	148	76.3
Heavy	610	27.6	36	29.8	836	25.9	46	23.7
Physical activity								
Often, sometimes	875	40.0	39	32.0	1608	49.6	88	44.2
Never	1312	60.0	83	68.0	1632	50.4	111	55.8
BMI, Body mass index								
18.5≦BMI<25.0	1685	75.1	90	70.3	2386	73.1	144	70.9
BMI<18.5	70	3.1	11	8.6	158	4.8	25	12.3
25.0≦BMI	490	21.8	27	21.1	718	22.0	34	16.7
Chronic diseases								
No	1568	71.4	55	44.7	1975	61.0	85	42.7
Yes	628	28.6	68	55.3	1264	39.0	114	57.3

Table 2 shows survival status for this cohort. After excluding the first year deaths, there were 131 deaths (5.7%) among the 2,282 rural subjects and 207 deaths (6.2%) among the 3,315 urban subjects during the 1993 to 2000 follow-up period. There were 42 (1.8%) and 102 (3.1%) censored cases in the rural and urban groups, respectively (Table. 2). These small numbers were considered to have negligible effects on the results of this study^17^^)^.

**Table 2.** Number of subjects, censored (%) and deaths (%) in the two cohorts: rural and urban.

	Rural		Urban	
	
	Total	Without early one-year-death cases	Total	Without early one-year-death cases
No. of subjects	2294	2282	3333	3315
No. of censored (%)	42(1.8)	42(1.8)	102(3.1)	102(3.1)
No. of deaths (%)	143(6.2)	131(5.7)	225(6.8)	207(6.2)
Person-year of follow-up	17157.94	17152.22	22059.95	22050.39

Table 3 presents age-adjusted relative risks for mortality in the rural and urban groups. With respect to sociodemogaphic variables, self-employed and low educated subjects had a significantly increased risk of mortality in the rural group. Educational background was not significantly associated with mortality in the urban group, though self-employed subjects had a significantly decreased mortality risk. In terms of social network variables, marital status (Marriage), participation in activities (Participation) and enjoying good fellowship with neighbors (Neighborhood) were significantly associated with mortality in the rural groups. Marital status (Marriage), household size (Household) and participation in activities (Participation) were significantly associated with mortality in the urban groups. Number of meeting close relatives (Relatives), having reliable friends (Friends) and going to any religious services (Religion) were not significantly associated with age-adjusted relative risks. Subjects without a spouse (Marriage) and those who did not participate in activities (Participation) had a significantly increased risk of mortality for both groups as compared with married subjects and those who participated in activities. Subjects who did not enjoy good fellowship with their neighbors (Neighborhood) had a significantly lower relative risk than those who did in the rural group, though there were no significant associations in the urban group. Subjects with a larger household size (Household) had a significantly lower relative risk than those with a smaller household size in the urban group, despite the lack of any significant associations in the rural group. In regard to lifestyle variables, smoking habit and BMI were significantly associated with mortality in the rural group. On the other hand, physical activity and BMI were significantly associated with mortality in the urban groups. Alcohol consumption showed no significant association with age-adjusted relative risks. Low BMI (less than 18.5) was associated with a significantly increased risk of mortality for both groups as compared to medium BMI (18.5 to less than 25.0). Current and former smokers had significantly higher relative risks of mortality than non-smokers in the rural group but there were no significant associations in the urban group. Subjects who did not exercise had a significantly higher relative risk than those who often or sometimes exercised in the urban group, despite there being no significant associations in the rural group. Chronic diseases and depressive status were both associated with significantly increased risks of mortality for both groups.

**Table 3.** Age-adjusted relative risk (RR) with 95% confidence interval (CI) for deaths from all causes by Cox proportional hazards model in the two cohorts: rural and urban.

Variable	Rural		Urban	
	
	RR	(95% CI)	RR	(95% CI)
Age				
	1.11	(1.08, 1.13)*	1.09	(1.07, 1.11)*
Occupation				
Salaried worker	1.00		1.00	
Self-employed	1.93	(1.24, 2.98)*	0.72	(0.53, 0.98)*
Agriculture and forestry	1.07	(0.68, 1.68)	0.83	(0.20, 3.34)
No occupation	1.47	(0.62, 3.48)	0.98	(0.58, 1.67)
Education				
Junior college and college or higher	1.00		1.00	
Except above	4.08	(1.30, 12.85)*	1.28	(0.89, 1.84)
Marriage				
Married	1.00		1.00	
Except above	1.73	(1.06, 2.80)*	1.65	(1.13, 2.42)*
Household				
Per each additional person	0.96	(0.87, 1.05)	0.86	(0.77, 0.95)*
Relatives				
Often, sometimes	1.00		1.00	
A few, never	0.88	(0.57, 1.35)	1.06	(0.75, 1.50)
Friends				
Yes	1.00		1.00	
No	0.95	(0.66, 1.39)	0.93	(0.70, 1.23)
Participation				
Yes	1.00		1.00	
No	1.52	(1.05, 2.21)*	1.79	(1.34, 2.40)*
Religion				
Yes	1.00		1.00	
No	0.80	(0.37, 1.72)	0.90	(0.52, 1.55)
Neighborhood				
Yes	1.00		1.00	
No	0.66	(0.46, 0.94)*	1.13	(0.84, 1.52)
Smoking habit				
Never	1.00		1.00	
Former	2.46	(1.40, 4.32)*	0.72	(0.45, 1.17)
Current	1.80	(1.08, 2.98)*	1.35	(0.97, 1.89)
Alcohol consumption				
Never, light	1.00		1.00	
Heavy	1.42	(0.95, 2.10)	1.02	(0.73, 1.43)
Physical activity				
Often, sometimes	1.00		1.00	
Never	1.26	(0.86, 1.85)	1.39	(1.05, 1.84)*
BMI, Body mass index				
18.5≦BMI<25.0	1.00		1.00	
BMI<18.5	2.68	(1.43, 5.02)*	2.06	(1.34, 3.16)*
25.0≦BMI	1.21	(0.79, 1.87)	0.84	(0.58, 1.22)
Chronic diseases				
No	1.00		1.00	
Yes	2.36	(1.64, 3.38)*	1.61	(1.21, 2.14)*
Depression				
Per each additional scaled score	1.08	(1.04, 1.13)*	1.05	(1.02, 1.09)*

* p<0.05

Table 4 presents multivariate relative risks identified using the Cox proportional hazards model adjusted simultaneously for all variables; age, occupation, educational background, marital status (Marriage), household size (Household), number of meeting close relatives (Relatives), having reliable friends (Friends), participation in activities (Participation), going to any religious services (Religion), enjoying good fellowship with neighbors (Neighborhood), smoking habit, alcohol consumption, physical activity, BMI, chronic diseases, and depressive status. Tendencies were similar to age-adjusted relative risks. Educational background, marital status (Marriage), enjoying good fellowship with neighbors (Neighborhood), and chronic diseases remained as significant as the associations in the rural group. Participation in activities (Participation), BMI, and chronic diseases also remained as significant as the associations in the urban group. The multivariate relative risk for low educated subjects was 4.4 (95%CI: 1.1-18.2) as compared to high educated subjects in the rural group. Subjects without a spouse (Marriage) had an increased risk of mortality (RR=2.4; 95%CI: 1.2-4.5) in the rural group. Subjects not enjoying good fellowship with their neighbors (Neighborhood) had a decreased risk of mortality (RR=0.58; 95%CI: 0.35-0.97) in the rural group. It was surprising that enjoying good fellowship with neighbors (Neighborhood) was adversely associated with mortality. The multivariate relative risk of subjects who did not participate in activities (Participation) was 1.6 (95%CI: 1.1-2.4) as compared to subjects who participated in activities in the urban group. Four other social network variables: household size (Household), number of meeting close relatives (Relatives), having reliable friends (Friends), and going to any religious services (Religion), were not associated with mortality. These results provided estimates of the independent associations of marital status (Marriage), enjoying good fellowship with neighbors (Neighborhood) and participation in activities (Participation), as social network factors, with mortality risk. With respect to lifestyle variables, only BMI (less than 18.5) was associated with a significantly increased risk of mortality (RR=2.1; 95%CI: 1.2-3.5) as compared to medium BMI (18.5 to less than 25.0) in the urban group but there were no significant associations in the rural group. Other lifestyle variables, such as smoking habit, alcohol consumption and physical activity, were not significantly associated with mortality for both groups.

**Table 4.** Multivariate relative risk (RR) with 95% confidence interval (CI) for deaths from all causes by Cox proportional hazards model in the two cohorts: rural and urban.

Variable	Rural		Urban	
	
	RR	(95% CI)	RR	(95% CI)
Age				
	1.09	(1.06, 1.13)*	1.09	(1.06, 1.12)*
Occupation				
Salaried worker	1.00		1.00	
Self-employed	1.28	(0.72, 2.27)	0.80	(0.55, 1.15)
Agriculture and forestry	0.72	(0.37, 1.39)	1.93	(0.46, 8.05)
No occupation	1.47	(0.54, 3.98)	0.94	(0.49, 1.80)
Education				
Junior college and college or higher	1.00		1.00	
Except above	4.39	(1.06, 18.21)*	1.07	(0.69, 1.64)
Marriage				
Married	1.00		1.00	
Except above	2.35	(1.22, 4.53)*	1.12	(0.64, 1.99)
Household				
Per each additional person	0.95	(0.82, 1.09)	0.87	(0.76, 1.00)
Relatives				
Often, sometimes	1.00		1.00	
A few, never	0.62	(0.35, 1.11)	0.97	(0.64, 1.46)
Friends				
Yes	1.00		1.00	
No	0.80	(0.47, 1.35)	0.73	(0.50, 1.05)
Participation				
Yes	1.00		1.00	
No	1.60	(0.95, 2.71)	1.61	(1.10, 2.37)*
Religion				
Yes	1.00		1.00	
No	1.10	(0.34, 3.60)	0.84	(0.44, 1.60)
Neighborhood				
Yes	1.00		1.00	
No	0.58	(0.35, 0.97)*	1.00	(0.69, 1.45)
Smoking habit				
Never	1.00		1.00	
Former	2.02	(0.97, 4.23)	0.77	(0.45, 1.32)
Current	1.23	(0.63, 2.40)	1.15	(0.77, 1.72)
Alcohol consumption				
Never, light	1.00		1.00	
Heavy	1.51	(0.88, 2.60)	1.22	(0.82, 1.82)
Physical activity				
Often, sometimes	1.00		1.00	
Never	1.16	(0.67, 1.98)	1.14	(0.80, 1.64)
BMI, Body mass index				
18.5≦BMI<25.0	1.00		1.00	
BMI<18.5	1.87	(0.78, 4.48)	2.05	(1.21, 3.47)*
25.0≦BMI	0.86	(0.45, 1.63)	0.76	(0.47, 1.23)
Chronic diseases				
No	1.00		1.00	
Yes	1.71	(1.05, 2.80)*	1.47	(1.03, 2.10)*
Depression				
Per each additional scaled score	1.07	(1.00, 1.15)	1.01	(0.96, 1.06)

* p<0.05

## DISCUSSION

### Effects of sociodemographic factors on mortality

#### Occupation

Ohta et al.^15^^)^ reported that the self-employed men in a rural area felt that their job was hard, had higher job satisfaction, and had slightly more subjective physical and mental complaints, while blue-collar workers had the poorest perceived health with many complaints in the rural area. In contrast, Cavelaars et al.^23^^)^ showed self-employed men to generally be healthier than the average population with respect to morbidity indicators including perceived health, long-term disabilities, chronic conditions and any long-standing health problem. The present findings indicate self-employed rural men to have a significantly increased age-adjusted mortality risk as compared to salaried workers, while the other two occupations did not have significantly higher risk. On the other hand, self-employed men in the urban group had a significantly decreased age-adjusted mortality risk as compared to salaried workers, while other occupations showed no significant associations. The multivariate model showed occupation to not be significantly associated with mortality in either rural or urban groups. Although these findings might indicate that the impact on mortality in self-employed men differs between rural and urban groups, the reason remains unclear. However, Hart^24^^)^ reported that the major explanation for occupational mortality differences should be sought in socioeconomic factors such as wealth and income, housing, educational and employment opportunities, and behavioral characteristics. Judging from the lack of an association with multivariate mortality risk in terms of occupation, potential confounding factors in the rural group may differ from those in the urban group. In regard to this point, explanations for these rural-urban differences merit further examination.

#### Education

Those with low education in the rural group had a significantly increased age-adjusted and multivariate mortality risk. Educational levels were not significantly related to mortality risk in the urban group. Smith et al.^25^^)^ reported that rural men were exposed to greater risk in association with lower educational levels, low income, and loss of a spouse, especially through divorce or separation. Some studies also showed low education to be associated with high mortality^26^^-^^28^^)^, though regional differences were not taken into account. Schrijvers et al.^28^^)^ concluded that the association between educational level and all-cause mortality was largely explained by a differential distribution of behavioral (alcohol, smoking, BMI, physical activity) and material (financial problems, employment status, income proxy) factors across educational groups. In this study, the relative risk for low educated subjects in the rural group was obviously higher than that for urban subjects. One possible interpretation is that differences between rural and urban may reflect inequality of income distribution which is depended on educational background. According to second wave survey in Komo-Ise 2000, average household income by area groups and educational levels showed that high educated men in the rural group tended to have higher average household income than those in the urban group. In contrast, low educated men in the rural group tended to have lower average household income than those in the urban group. Bigger income difference among rural men may account for the difference in mortality risk between rural and urban^29^^)^. However, no adjustment was made for economic status in the multivariate model. Education was considered to be one of the most important factors explaining rural-urban differences.

### Effects of social network factors on mortality

#### Marriage

In this study, though subjects without a spouse (Marriage) had a significantly increased age-adjusted mortality risk in both groups, this relationship remained in the rural group even after adjusting for potential confounding factors. This suggests that marital status is an independent risk factor in rural subjects. A few reports have referred to area differences in terms of marital status and mortality, though a number of studies have repeatedly shown increased mortality in non-married persons, i.e., widows and widowers, divorced, and the never married, as compared with married persons^26^^,^^30^^)^. According to Smith et al.^25^^)^, the adverse effect of divorce and separation among rural residents was explained as divorce or separation possibly being more psychologically and socially damaging because a rural community regards durable marriages as generally meeting desired standards of social behavior as compared to an urban community where marriage failure is more common. Our findings are partially consistent with theirs, though the reason for the difference has not yet been systematically determined. The impact of marital status to mortality may reflect sociocultural difference between rural and urban. One speculation is that men among the rural group might tend to be more dependent on their spouse than those among the urban group, judging from smaller and denser personal networks among the rural group which consisted of mainly family and kinship^31^^)^. The death rates of divorced, widowed, and never-married men might differ in our present study, but there were too few deaths to examine them separately according to each marital status category.

#### Participation

Lack of participation in activities (Participation) was associated with a significantly increased age-adjusted mortality risk in both groups. In a multivariate model, this relationship was preserved only in the urban, not in the rural group. However, participation in activities (Participation) was regarded as an important predictor of mortality in middle-age men irrespective of group, as the relative risks were very similar (1.60 rural vs. 1.61 urban). Welin et al.^32^^)^ reported high levels of social, home, and outside home activities to protect middle-age men from premature deaths. Social activities had a particularly strong association with mortality. Bygren et al.^33^^)^ also pointed out the importance of attending cultural events in terms of protecting against premature deaths, though there were areas of overlap among several variables. Sugisawa et al.^13^^)^ revealed social participation of Japanese people who were elderly rather than middle-aged to produce direct and indirect effects on morality linked with functional status and self-rated health, when physical health and health behavior were hypothesized to mediate the effects of social networks and social support on mortality. According to Sasazawa et al.^16^^)^, physical activity was associated with better perceived health and social network in a cross-sectional survey. These reports indicated that not only the direct effect but also the indirect effect on mortality should be clarified to determine the impact of social network on mortality in middle-aged Japanese men.

#### Friends, Relatives and Religion

Number of meeting close relatives (Relatives), having reliable friends (Friends), and going to any religious services (Religion) were not found to have statistically significant effects on the risk of death in either group. These findings are considered to reflect a lack of rural-urban differences. Previous studies indicated inadequate social relations as indicated by relatively few social ties and a low level of social support to be associated with an excess mortality risk in Western developed countries^5^^,^^34^^,^^35^^)^. Berkman et al.^5^^)^ reported the more intimate ties of marriage and contact with friends and relatives to be stronger predictors than the ties of church and group membership. As these findings might reflect the Western sociocultural context, there is no guarantee that the same results would be obtained in a Japanese population. Indeed, findings from previous studies of elderly Japanese were much less consistent with findings in Western countries. Fujita et al.^36^^)^ reported that there was no statistically significant association between social network and mortality and no differences among three socioeconomically diverse communities. According to Sugisawa et al.^13^^)^ social participation had a significant effect on the risk of death in elderly Japanese while marital status and social contacts were not statistically associated with mortality. The impact of marital status on mortality was greatest in the younger age group, diminishing in the older groups^27^^)^. As to this point, these findings were considered to be similar to our present results except for enjoying good fellowship with neighbors (Neighborhood) among rural men.

#### Household

Household size (Household) was not associated with statistically significant effects on mortality in age-adjusted and multivariate models in the rural group. On the other hand, larger household size (Household) related significantly to lower age-adjusted mortality risk in the urban group, though this relationship was come near to preserving after adjusting for potential confounding factors (the observed significance level of the test was 0.0501). Sorlie et al.^26^^)^ reported quite similar findings, i.e., household size was not significantly associated with multivariate adjusted mortality risk for middle-age men, while large household size decreased age-adjusted mortality risks. Judging from the multivariate model, our findings suggest the difficulty to conclude rural-urban differences with respect to household size (Household).

#### Neighborhood

One of our notable findings is the adverse association between enjoying good fellowship with neighbors (Neighborhood) and mortality, though the interpretation and explanation of this association is unclear. Beggs et al.^31^^)^ reported the characteristics of personal networks for rural residents, that is, ties were of greater intensity, were based more on kinship and neighborhood and were smaller and denser than personal networks for urban residents. These findings may indicate that rural residents are rooted and have relations of longer duration to community, family, and neighborhood. Shimada et al.^37^^)^ pointed out the characteristics of rural societies in Japan that had formal neighborhood relationships. In a non-urbanized rural society, well-organized small groups, which are called *Kumi* or *Han*, contribute to provide support for each group member in daily life, for example taking turns sweeping, and cooperating and participating in marriage, funeral and ancestral ceremonies, and so on. Each member was required to help others, to take part in their duties, and to play their roles regardless of their personal desires. The formal neighborhood relationships did not always involve absolute intimacy, though rural people tended to have stronger neighborhood relationships than those of relatives. In other words, this neighborhood relationship in the rural society sometimes may force residents to be under high demands and low control that produce stress to them. As a result, they who are involved in this situation may be more likely to have health problems. Although this hypothesis has been confirmed that the situation under high demands and low control related to incidence and mortality of chronic disease among office workers^38^^)^, we have no evidence among residents as far as we know. According to Katsura et al.^39^^)^, Japanese middle-aged rural residents recognized relatively trivial affairs as stressful in routine daily human relationships, such as the relation to one’s neighborhood, as well as bereavement of their spouse or a close family member. Miller et al.^40^^)^ showed an interesting relationship between the number of acquaintances and a number of psychological and physical symptom scores. Persons with a lot of acquaintances did not exactly have the lower the scores, i.e., persons with “some acquaintances” had the lowest scores. These reports indicated two important limitations to our study, though we suspect that enjoying good fellowship with neighbors (Neighborhood) increased the risk of mortality among rural residents. One possibility is investigating not only the quantity but also the quality of social relationships and the other is estimating the effect of stress quantitatively to adjust for confounding factors in multivariate models. Social relationships are considered to consist of both social networks and social support. Social networks are often defined as the quantity or structural aspects of relationships. On the other hand, social support is commonly explained as the quality or functional aspects of relationships. Cohen et al.^41^^)^ showed social support to affect the physical and mental health of the individual via two main pathways. One is a direct positive effect and the other is a buffer or modifier of the effects of psychosocial and physical stress. The stress-buffering model proposes that there is an interaction of stress and social relations on health. Stress has an adverse effect on health and wellbeing only for those with inadequate social relations. With respect to relations with neighbors (Neighborhood) among rural men, we must pay attention not only to its interpretation but also give greater consideration to this issue.

### Effects of lifestyle factors on mortality

#### BMI

The relation between body weight and mortality remains controversial. Some prospective studies have shown the relationship between BMI and mortality to be represented by a U-shaped or L-shaped curve in which the risk was increased among the very heavy and the very lean or among only the very lean^2^^,^^17^^,^^42^^)^. On the other hand, Lee et al.^43^^)^ reported a J-shaped relation between BMI and mortality and concluded that there was no evidence of excess risk among lean men. In this study, the lean group (BMI<18.5) had a significantly increased age-adjusted mortality risk in both groups. In the urban group especially, this relationship remained after adjusting for potential confounding factors. In the rural group, the excess risk (RR=1.87) did not reach statistical significance. With respect to BMI, our findings were considered to reflect the lack of a difference between rural and urban groups. Manson et al.^44^^)^ emphasized the importance of assessing subclincal conditions related to weight loss at baseline. Although the subjects were asked about their health conditions including chronic diseases at baseline and we excluded deaths within the first year from this analysis, these steps might not have been enough to eliminate the influence of subclincal conditions.

#### Smoking and Physical activities

Though current and former smokers in the rural group had a significantly increased age-adjusted mortality risk, this relationship did not remain after adjusting for sociodemographic, social network, and other lifestyle factors. On the other hand, current and former smokers in the urban group did not show significant excess risk in either model. In contrast to smoking habit, urban subjects who did not exercise had a significantly increased age-adjusted mortality risk, though this was not demonstrated by the multivariate model. Rural subjects who did not exercise did not show a significant association with age-adjusted and multivariate mortality risks. Although studies have repeatedly shown smoking habit and physical activity to be independent risk factors for mortality^1^^,^^4^^)^, our findings were not consistent with those of previous studies. However, several studies have reported smoking habit and physical activity to be associated with educational level^16^^,^^45^^,^^46^^)^. This might suggest that regional differences to be explained by educational level rather than lifestyle factors, such as smoking habit and physical activity. Low educated people with adverse lifestyle factors should be targeted for prevention of premature deaths by means of public health measures.

### Limitations of this study

Some limitations of this study should be taken into account. First, this study focused only on men because data from the Tecumseh Community Health Study indicated that social networks might be less important for women than for men^9^^)^. Furthermore, fewer women than men died, such that there were too few to conduct multivariate analyses. Given these two issues, we did not analyze the data for women in this study. Second, our baseline data did not include objective data such as physical and physiological examination results, and laboratory data, as our baseline survey was conducted using a self-administered questionnaire. Third, it must be kept in mind that other confounding factors may remain, though we took several potential confounding factors into consideration. As mentioned earlier, it must be emphasized again that the effects of stress are not included in this study, despite the importance of social relationships as well as mortality^14^^,^^41^^)^. Fourth, this study examined all explanatory and control variables obtained at the time the baseline data were obtained. During the approximately seven year follow-up periods, these variables may have changed with the passage of time. The impacts of such changes on the risk of mortality should be taken into account^47^^)^. Fifth, we cannot completely rule out the possibility of the indirect effects of social networks on mortality, as we focused on direct effects in this study^22^^)^. Therefore, the possibility exists that men without a spouse (Marriage) and/or participation in activities (Participation) develop a less healthy lifestyle than others.

Despite these limitations, this research has provided significant new knowledge for Japanese middle-aged men, regarding rural-urban differences in associations of sociodemographic, social network, and lifestyle factors to mortality. Participation in activities (Participation) could be important for preventing premature death in middle-age Japanese men. Rural-urban differences were identified for education, martial status (Marriage) and enjoying good fellowship with neighbors (Neighborhood) which had strong impacts on mortality in the rural group, though differences between the rural and urban groups in lifestyle factors and social network factors including household size (Household), number of meeting close relatives (Relatives), having reliable friends (Friends), and going to any religious services (Religion) were not significant.
